# Roosting ecology of endangered plant‐roosting bats on Okinawa Island: Implications for bat‐friendly forestry practices

**DOI:** 10.1002/ece3.8101

**Published:** 2021-09-17

**Authors:** Jason H. Preble, Christian E. Vincenot, Kazuhiko Saito, Nobuhito Ohte

**Affiliations:** ^1^ Department of Social Informatics Graduate School of Informatics Kyoto University Kyoto Japan; ^2^ Island Bat Research Group (IBRG) Kyoto Japan; ^3^ Kansai Research Center, Forestry and Forest Research Products Institute Fushimi Kyoto Japan

**Keywords:** Chiroptera, foliage‐roosting, forest management, reproduction, roost switching, tree‐roosting

## Abstract

Roosting information is crucial to guiding bat conservation and bat‐friendly forestry practices. The Ryukyu tube‐nosed bat *Murina ryukyuana* (Endangered) and Yanbaru whiskered bat *Myotis yanbarensis* (Critically Endangered) are forest‐dwelling bats endemic to the central Ryukyu Archipelago, Japan. Despite their threatened status, little is known about the roosting ecology of these species and the characteristics of natural maternity roosts are unknown. To inform sustainable forestry practices and conservation management, we radio‐tracked day roosts of both species in the subtropical forests of Okinawa's Kunigami Village District. We compared roost and roost site characteristics statistically between *M. ryukyuana* nonmaternity roosts (males or nonreproductive females), maternity roosts, and all *M. yanbarensis* roosts. Generalized linear models were used to investigate roost site selection by *M. ryukyuana* irrespective of sex and age class. Lastly, we compiled data on phenology from this and prior studies. Nonreproductive *M. ryukyuana* roosted alone and primarily in understory foliage. *Murina ryukyuana* maternity roosts were limited to stands >50 years old, and ~60% were in foliage. *Myotis yanbarensis* roosted almost entirely in cavities along gulch bottoms and only in stands >70 years old (~1/3 of Kunigami's total forest area). *Murina ryukyuana* maternity roosts were higher (4.3 ± 0.6 m) than conspecific nonmaternity roosts (2.3 ± 0.5 m; *p* < .001) and *M. yanbarensis* roosts (2.7 ± 0.5 m; not significant). Model results were inconclusive. Both species appear to be obligate plant roosters throughout their life cycle, but the less flexible roosting preferences of *M. yanbarensis* may explain its striking rarity. To conserve these threatened bats, we recommend the following forestry practices: (a) reduce clearing of understory vegetation, (b) refrain from removing trees along streams, (c) promote greater tree cavity densities by protecting old‐growth forests and retaining snags, and (d) refrain from removing trees or understory between April and July, while bats are pupping.

## Graphical abstract

We investigated the roosting ecology of two threatened island bats: the Ryukyu tube‐nosed bat *Murina ryukyuana* and Yanbaru whiskered bat *Myotis yanbarensis*. *Murina ryukyuana* roosted primarily in understory foliage, using tree cavities more often as maternity roosts, while *M. yanbarensis* roosted almost entirely in cavities along gulch bottoms and only in stands >70 years old. We provide recommendations for bat‐friendly forestry practices based on our results.

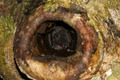

## 要約

コウモリのねぐらに関する生態学的な情報はコウモリの保全とそれを考慮する森林の施業のために重要である。リュウキュウテングコウモリ*Murina ryukyuana*（絶滅危惧）とヤンバルホオヒゲコウモリ*Myotis yanbarensis*（絶滅寸前）は森林性コウモリで、日本の琉球列島中部における固有種である。絶滅の恐れがある種にもかかわらず、これらの種のねぐらに関する生態については情報が不足しており、自然条件下における母子集団のねぐらの特徴は明らかになっていない。持続可能な森林施業と保全に関わる情報を提供するために、沖縄島国頭村における亜熱帯林で両種の昼間のねぐらの場所や特徴を、ラジオテレメトリー法によって個体を追跡することによって調査した。リュウキュウテングコウモリの非母子集団ねぐら（雄または非繁殖雌）、母子集団ねぐら、及び全てのヤンバルホオヒゲコウモリのねぐらの間で、ねぐらとねぐらの場所の特徴を統計的に比較した。一般化線形モデルを用いて、性別や年齢階級を考慮しない条件でリュウキュウテングコウモリによるねぐら場所の選好性を調査した。最後に、本研究と先行研究から、ねぐらに関わるフェノロジーに関するデータを収集・整理した。非生殖のリュウキュウテングコウモリは単独で、主に下層植生の葉をねぐらとして利用した。リュウキュウテングコウモリの母子集団のねぐらとしての利用は林齢50年以上の林分に限定され、約60％が葉であった。ヤンバルホオヒゲコウモリはほぼ完全に谷底の樹洞をねぐらとして利用し、ねぐらの形成は林齢70年以上の林分に限られていた（国頭村における森林面積の約1／3）。リュウキュウテングコウモリの母子集団ねぐらは同種の非母子集団ねぐら（2.3 ± 0.5 m; p < .001）とヤンバルホオヒゲコウモリのねぐら（2.7 ± 0.5 m; not significant）より高い位置にあった（4.3 ± 0.6 m）。モデルによる推定結果は決定的ではなかった。両種はライフサイクルの間ずっと偏性的な植物性コウモリであるようだが、ヤンバルホオヒゲコウモリの選好は柔軟性が低く、このことが本種の際だった希少性を説明する可能性がある。これらの絶滅危惧種であるコウモリを保全するためには、以下の森林施業が提案できる：（a）下層植生の除去を少なくすること、（b）渓流沿いの樹木の伐採を控えること、（c）高林齢の森林を保存し、枯死木も除去せず、樹洞の密度を確保すること、（d）コウモリが子育てしている時期である4月〜7月の間に、樹木と下層植生の除去を控えることである。

## 1 INTRODUCTION

Understanding species‐specific roosting requirements is critical to effectively conserving bats (Altringham, 2011). Bats are of high conservation concern, with over a third of the world's >1,200 assessed bat species considered threatened or data deficient (Frick et al., 2019). Bats also serve as bioindicators (De Conno et al., 2018; Jones et al., 2009) and provide key ecosystem services including pollination, seed dispersal, and the regulation of insect populations (Kunz et al., 2011). Protecting roosting habitats, where bats rest and reproduce (Racey & Entwistle, 2000), is essential to restoring and maintaining bat populations and communities. Maternity roosts are of particular interest to conservationists (Nad'o & Kaňuch, 2015) because reproductive females are important to population viability and often more sensitive to disturbance (Pryde et al., 2005). Although bats are often associated with underground roosts, many species roost in trees or other plants. Here, we use the term “plant‐roosting” to refer to bats that roost in any part of a tree (commonly referred to as “tree‐roosting”) or any other plants (ferns, shrubs, bamboo, pitcher plants, etc.). This includes bats that roost in woody cavities (“cavity‐roosting”) or foliage (“foliage‐roosting”). These plant‐roosting bats can be particularly difficult to study as they often roost alone or in relatively small colonies in difficult to find roosts and switch roosts often (Lewis, 1995).

Forestry‐driven changes to forest composition and stand age affect bats according to their roosting and foraging ecology (Russo et al., 2016). Cavity‐roosting bats usually roost in larger diameter trees and snags (Kalcounis‐Rüppell et al., 2005; Nad'o & Kaňuch, 2015). If ecological values are not considered, foresters are incentivized to remove such trees for sale, to improve remaining tree regeneration, or to reduce safety hazards associated with falling debris (Guldin et al., 2007). Less is known about foliage‐roosting bats, but some may also prefer larger trees in mature forests (Carter & Menzel, 2007). Studies generally recommend preserving old‐growth stands or retaining mature trees and snags in mixed‐age stands since many tree‐roosting bats appear reliant on these habitats (e.g., Burgar et al., 2015; Perry & Thill, 2007; Webala et al., 2010). Thinning trees or clearing understory foliage to promote the growth of remaining trees may benefit open‐space and edge‐foraging bats while negatively impacting clutter‐foraging species (e.g., Patriquin & Barclay, 2003, Carr et al., 2020). For example, species including the open‐space foraging *Lasiurus seminolus* prefer roosting in relatively open forests (Perry et al., 2007), while the clutter‐foraging *Barbastella barbastellus* prefer roosts in unmanaged forests with dense understory (Russo et al., 2004). Forestry practices can reduce negative impacts to bats, or sometimes even benefit bats, by taking their ecological requirements into account (Hayes & Loeb, 2007). However, forestry managers often lack sufficient information to adopt more bat‐friendly practices (Law et al., 2016). Furthermore, though general trends exist, the interactions between various forestry practices and different bat species may differ between regions and forest types.

Most of our knowledge concerning plant‐roosting bat ecology comes from research on tree‐roosting bats in North America, Europe, and Australia (e.g., Lacki et al., 2007; Law et al., 2016), while data from other regions are sparse (Kingston, 2010; Racey, 2015). Threatened but poorly understood species are unfortunately common among the bats of Asia and the Pacific Islands (Wiles & Brooke, 2009, Conenna et al., 2017). The genus *Myotis* is the most represented in tree‐roosting bat studies thanks to extensive research on the relatively small proportion of *Myotis* species native to Western countries (Nad'o & Kaňuch, 2015), but the majority of *Myotis* in other regions are poorly studied (Moratelli & Burgin, 2019). Similarly, few *Murina* species have been the subject of roosting studies (e.g., Fukui et al., 2012; Schulz & Hannah, 1998), and the proportion grows increasingly small when considering the remarkable rate of recent species discoveries in this genus (Yu et al., 2020). In Japan, ecological research concerning endemic plant‐roosting bats has been limited despite most being threatened (Preble et al., 2021).

The Ryukyu tube‐nosed bat *Murina ryukyuana* and Yanbaru whiskered bat *Myotis yanbarensis* are rare forest‐dwelling bats endemic to three islands in Japan's central Ryukyu Archipelago: Okinawa, Tokunoshima, and Amami Ōshima (Maeda et al., 2001, 2002; Maeda & Matsumura, 1998). *Murina ryukyuana* and *M. yanbarensis* are considered “Endangered” and “Critically Endangered,” respectively, by the IUCN and Japanese Ministry of the Environment (Fukui & Sano, 2019a, 2019b; Ministry of the Environment, 2014). These assessments consider these species reliant on intact mature forests. However, ecological information is limited, especially concerning *M. yanbarensis* (Funakoshi et al., 2013, 2019), and research has been practically absent on Okinawa. *Murina ryukyuana* have been found roosting mostly in understory foliage, and only seven *M. yanbarensis* roosts have been reported, five in tree cavities and two in culverts (Asari et al., 2021; Funakoshi et al., 2019; Watari & Funakoshi, 2013). Neither species is likely to hibernate, but winter records are sparse. Notably, no natural maternity day roosts have been reported for either species, although maternity colonies of *M. ryukyuana* have used artificially placed dry leaves as day roosts (Funakoshi et al., 2013), and mothers with prevolant pups were observed night‐roosting in canopy foliage on Amami Ōshima (V. Dinets, unpublished). Both species have been identified as high research priorities because they are threatened, endemic, and poorly understood (Preble et al., 2021).

We radio‐tracked *M. ryukyuana* and *M. yanbarensis* on Okinawa over two years to clarify the roosting habits of these elusive bats and thereby inform conservation measures and bat‐friendly forestry practices. Specifically, we sought to (a) determine what types of roosts and roost sites these bats use to inform conservation of these habitats, (b) determine when and where maternity colonies form so forestry activities can avoid these sensitive roosts, and (c) compare roosting ecology between *M. ryukyuana* and *M. yanbarensis* to determine to what degree management strategies must be species‐specific. We were particularly interested in the influence of stand age as existing local biodiversity conservation plans prioritize the preservation of older forests (Okinawa Prefecture Department of Agriculture, Forestry, & Fisheries, 2013). Our primary hypotheses were that both species roost only within forests, that *M. ryukyuana* roosts flexibly in both foliage and cavities in stands of various ages, and that *M. yanbarensis* roosts only in cavities in old‐growth stands.

## 2 MATERIALS AND METHODS

### 2.1 Study site

**FIGURE 1:**
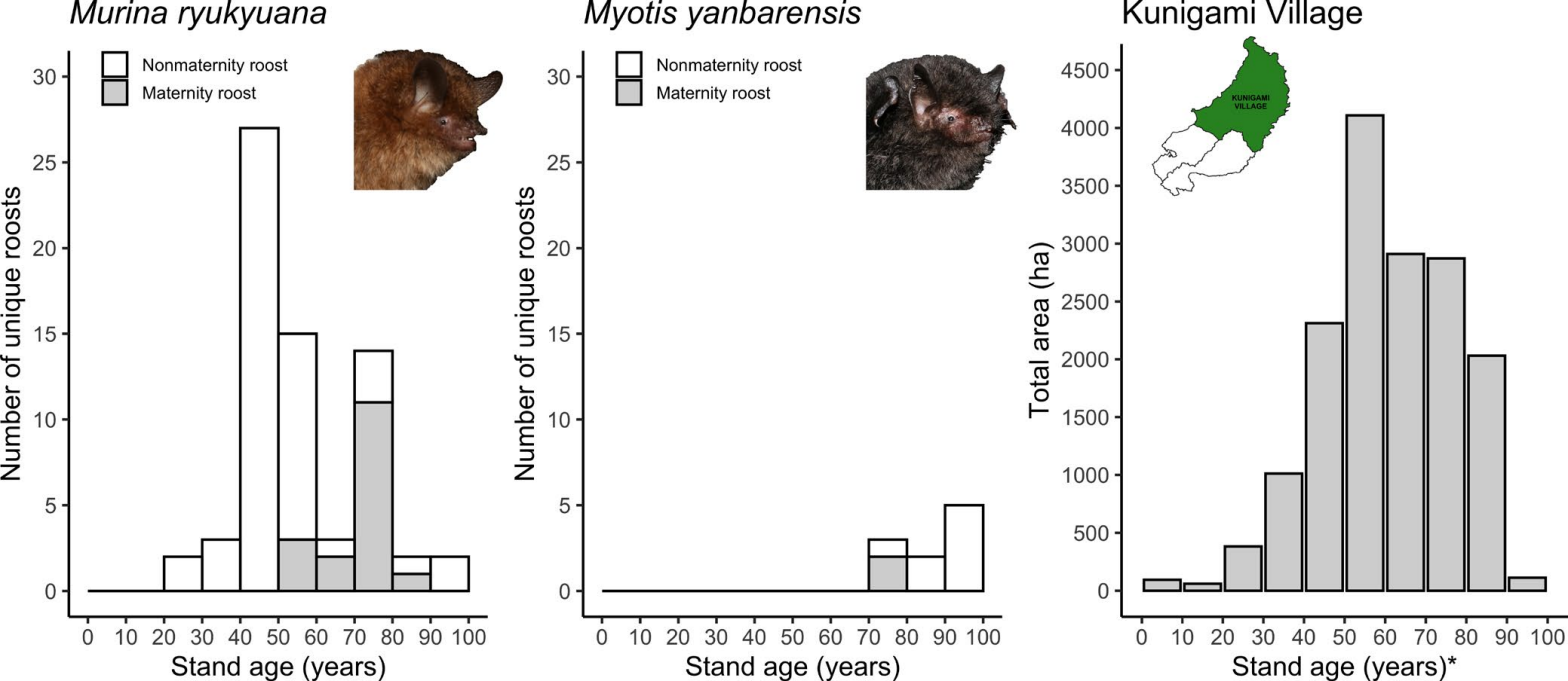
Left‐most histograms show stand age of unique *Murina ryukyuana* (*n* = 68) and *Myotis yanbarensis* (*n* = 10) roost sites (5 m radius plots around roosts) for which stand age data were available. Right‐most histogram shows total area (ha) by stand age within the Kunigami Village District to illustrate the distribution of stand ages potentially available within the study area. * Kunigami Village stand ages are measured from 2014, as reported in the most recently available local forest register, instead of 2020 because stand ages could not be updated across the whole district as they were for roost sites

Our study was conducted in the forests of the Kunigami Village District (26°45′N, 128°15′E), part of the Yambaru region of Okinawa, Japan. The low human population (~5,000 people) is concentrated around the coasts, and the interior of the roughly 20,000 ha area consists of low mountains (max. ~500 m altitude). Numerous streams weave through subtropical broadleaf forests dominated by *Castanopsis sieboldii* (Family Fagaceae). Yambaru is one of Japan's most biodiverse regions (Ito et al., 2000). Yambaru's forests have also served as a wood resource since the Ryukyu Kingdom period (15th–19th century). Heavy logging of secondary forests occurred during the 1950s postwar reconstruction period (Saito, 2011). Mature trees were targeted from the 1960s to 1990s, and since around 1990, forestry has greatly declined. As a result, Okinawa's northern forests are mostly of moderate age, with only 3% of total forest area <30 years old and 32% >70 years old (Figure 1). Large mature trees occur in low densities and are limited to old‐growth stands remaining in the Kunigami Village District and the US Military's Northern Training Area. Local forestry is government‐subsidized to stimulate the economy, and there is increasing pressure on the government to properly manage forests for biodiversity conservation (Ito et al., 2000; Sugimura, 1988), especially given the recent National Park and UNESCO natural World Heritage site designations.

### 2.2 Bat capture and radio tracking

We captured bats using a combination of harp traps, mist nets, and acoustic lures across 50 sites between September 2017 and September 2019. Capture devices were usually set perpendicular to flight paths over trails or streams. To increase capture rates, we broadcast synthesized stimuli based on social calls of unrelated species and conspecifics using Sussex Autobat acoustic lures (Hill & Greenaway, 2005). Captured females were categorized as nonreproductive, reproductive (pregnant or lactating), postlactating, or juvenile. Juveniles were identified by their lack of wing joint ossification (Brunet‐Rossinni & Wilkinson, 2009). We attached VHF radio transmitters (Holohil LB‐2X) to 17 *M. ryukyuana* (eight male and nine female) and ten *M. yanbarensis* (seven male and three female; Appendix S1) to identify roosts. We attached transmitters between each bat's shoulder blades using Pros‐Aide Adhesive (ADM Tronics Unlimited, Inc.). Transmitter weight was 3.3%–3.8% and 4.7%–5.4% of tracked *M. ryukyuana* and *M. yanbarensis* bodyweight, respectively.

We tracked bats to day roosts using a handheld radio receiver and Yagi antenna. When we could not visually confirm roosts, we triangulated their location. From May 2018 onwards, we used a thermal imager to improve visual searching (Pulsar Quantum XQ23V, Yukon Advanced Optics Worldwide). For each roost, we recorded roost type (foliage or cavity), roost height (to nearest 0.5 m), and roost plant species. Understory cover at each roost site (5 m radius plot surrounding the roost) was estimated visually to the nearest 5%. We recorded diameter at base height (DBH) for roost trees, tree ferns, and bamboo. When cavity roosts could be reached, we measured the entrance area and internal dimensions to calculate cavity volume following Sedgeley & O’Donnell (1999). Roosts were categorized as maternity roosts if they included multiple bats and juveniles, reproductive females, or postlactating females. We recorded the minimum number of individuals per roost using various methods including visual inspection, emergence counts, and rarely by capturing roosting bats.

### 2.3 Roost site comparisons

We compared roost type proportions between *M. ryukyuana* nonmaternity roosts, maternity roosts, and all *M. yanbarensis* roosts using Fisher's exact tests with Bonferroni corrections. Sample sizes were too low to assess differences between nonmaternity and maternity *M. yanbarensis* roosts. We used Kruskal–Wallis tests followed by Dunn's tests with Bonferroni corrections to compare roost height and the following roost site characteristics between the aforementioned groups: understory cover, canopy cover, canopy height, “southwestness” (SWness), slope, topographic position index (TPI), stream distance, and stand age. Stand age was calculated as years since the last clear‐cutting or substantial logging measured from 2020. Stand age estimates were based on local forest register data from 2014 and revised if necessary based on historical aerial photography (US Air Force 1944, 1946, 1962; Geospatial Information Authority of Japan 1973, 1977, 1989, 2002; Nakanihon Air Service 2011; NTT Geospace 2013). All other variables were derived from airborne laser scanning LiDAR data (Nakanihon Air Service 2011). LiDAR‐derived variables were calculated as the average value of 1‐m^2^ cells within a 5 m radius of the site center. Canopy cover was calculated as the proportion of cells within the site where the difference between elevation and vegetation height was >3 m. TPI was calculated using an inner and outer radius of 5 and 15 m, respectively. Stream distance (distance to the nearest stream) was calculated in ArcGIS based on a stream layer created using a minimum flow accumulation of 22,500 m^2^. Aspect was transformed to SWness following Beers et al. (1966), where southwest slopes, most commonly exposed to the sun, were given a value of 2 and northeastern slopes a value of 0. Prior roosting studies have used northness and eastness (e.g., Hammond et al., 2016), but we used SWness to reduce the number of covariates. Lastly, roost fidelity and maximum roost‐switch distance were calculated per species by averaging values per individual. Roost fidelity was calculated for each individual as the mean number of days before switching roosts. All analyses except for roost fidelity and roost‐switch distance calculations considered only unique roosts to avoid bias toward roosts used multiple times.

### 2.4 Murina ryukyuana roost site selection

We generated thirteen generalized linear models (GLM) reflecting eleven a priori hypotheses as to what characteristics influence roost site selection by *M*. *ryukyuana*, as well as a random model and global model (Table 1). Hypotheses included covariates from the literature known to affect roost selection by tree‐roosting bats. Higher snag density, tree diameter, tree height, and lower stream distance and canopy cover are generally preferred by tree‐roosting bats that use cavities (e.g., Fabianek et al., 2015; Nad'o & Kaňuch et al., 2015). Tree‐roosting bats that roost in foliage generally prefer higher canopy cover (e.g., Kalcounis‐Rüppell et al., 2005). Tree‐roosting bats have also been found to prefer warmer microclimates, such as lower canopies and south‐facing slopes (in the northern hemisphere) exposed to greater solar radiation (e.g., Hammond et al., 2016; Kerth et al., 2001; Law et al., 2016). We assumed higher stand ages to have higher snag densities and average tree diameters. Canopy height was used as a proxy for average tree height and SWness as a proxy for warmer microclimates. For each *M*. *ryukyuana* roost site, a random site was selected from within a 1 km buffer and similar site variables measured using GIS. The response variable for GLMs was site use (roost site = 1, random site = 0), and the explanatory variables included canopy height, SWness, slope, TPI, stream distance, and stand age. The quadratic term for stand age was also included as histograms suggested *M*. *ryukyuana* might prefer intermediate values. Canopy cover was not included as inclusion did not affect relative model performance, and only four roost sites and seven random sites had values <100%. We were unable to include understory cover as we did not have random site values. All explanatory variables were sufficiently uncorrelated (Spearman correlation coefficient <0.6). All covariates were centered before modeling. We used Akaike's information criterion corrected for small sample size (AICc) to assess model fit (Burnham & Anderson, 2002). We considered ∆AICc ≤ 4.0 to be supported by the data and examined well‐supported models for uninformative parameters (Arnold, 2010). Data were too scant to repeat this process for *M*. *yanbarensis*.

**TABLE 1:** Models for roost site selection by *Murina ryukyuana* including degrees of freedom (*K*), difference in AICc relative to the model most supported by the data (∆AIC_C_), Akaike weights (*w*), and Nagelkerke's *R* ^2^

Hypothesis	Model	*K*	∆AICc	*w_i_*	*R^2^*
Global model	All variables excluding stand age^2^	7	0.00	0.59	0.45
Warm‐tall‐old	~canopy height + stand age + SWness	4	1.97	0.22	0.40
Warm‐tall‐intermediate	~canopy height + stand age + stand age^2^ + SWness	5	2.38	0.18	0.41
Mature structure	~canopy height + stand age	3	9.08	0.01	0.33
Intermediate structure	~canopy height + stand age + stand age^2^	4	10.26	0.00	0.34
Typical tree cavity bat roost	~canopy height + stream distance + stand age	4	11.17	0.00	0.33
Solar radiation	~SWness + TPI	3	13.35	0.00	0.30
Wetter the better	~stream distance + SWness + TPI	4	15.45	0.00	0.30
Concealed habitats	~TPI	2	22.85	0.00	0.22
Typical foliage bat roost	~canopy height	2	26.25	0.00	0.19
Null	~1	1	47.07	0.00	0.00
Streams	~stream distance	2	47.78	0.00	0.01
Slopes	~slope	2	49.03	0.00	0.00

### 2.5 Phenology

To estimate the reproductive phenology of *M. ryukyuana* and *M. yanbarensis*, we compiled all records of reproductive females (pregnant or lactating) captured, maternity roosts, and female nonmaternity roosts from this and prior studies (Funakoshi et al., 2019; Maeda et al., 2001, V. Dinets, unpublished).

## 3 RESULTS

All 17 tracked *M*. *ryukyuana* were successfully relocated (Appendix S1), resulting in 141 day roost records (mean ± *SE*; 6.2 ± 0.6 roosts per individual). We visually confirmed 105 roosts representing 73 unique roosts—56 nonmaternity roosts and 17 maternity roosts. From six *M*. *yanbarensis* (four male and two female) relocated at least once, we visually confirmed 27 roosts (4.5 ± 1.1 roosts per individual). Ten *M. yanbarensis* roosts were unique, including two maternity roosts. One *M. yanbarensis* roost was used as both a nonmaternity roost and a maternity roost. Roost and roost site characteristics are summarized in Table 2.

**TABLE 2:** Summary of unique visually confirmed roost site and random site characteristics (mean ± *SE*)

Variable	*Murina ryukyuana*		*Myotis yanbarensis*	Random	Dunn's test comparisons		
	NM (*n* = 56)	M (*n* = 17)	All Myo (*n* = 10)	(*n* = 73)	NM v M	NM v Myo	M v Myo
Roost height (m)	2.3 ± 0.5	4.3 ± 0.6	2.7 ± 0.5	NA	***	n.s.	n.s.
Understory cover (%)	84.9 ± 2.2	86.5 ± 3.3	51.5 ± 8.5	NA	n.s.	**	**
Canopy cover (%)	99.2 ± 0.5	98.5 ± 1.5	100.0 ± 0.0	96.9 ± 1.6	n.s.	n.s.	n.s.
Canopy height (m)	14.6 ± 0.5	17.6 ± 0.8	16.8 ± 0.8	12.1 ± 0.5	**	n.s.	n.s.
Southwestness	1.49 ± 0.07	1.14 ± 0.13	1.22 ± 0.27	1.03 ± 0.08	*	n.s.	n.s.
Slope (˚)	30.7 ± 1.2	33.9 ± 2.6	29.6 ± 3.1	31.0 ± 1.2	n.s.	n.s.	n.s.
TPI	−0.71 ± 0.12	−0.77 ± 0.19	−1.39 ± 0.40	0.07 ± 0.11	n.s.	n.s.	n.s.
Stream distance (m)	58.7 ± 6.0	36.3 ± 7.7	7.2 ± 3.9	62.2 ± 5.6	n.s.	***	*
Stand age (yrs)	51.9 ± 1.8 (*n* = 51)	70.9 ± 2.0	86.7 ± 2.9	66.3 ± 2.2	***	***	n.s.
DBH (cm)	15.8 ± 7.8 (*n* = 5)	22.8 ± 4.8 (*n* = 13)	18.4 ± 3.7 (*n* = 8)	NA	NA	NA	NA
Entrance area (cm^2^)	25, 35 (*n* = 2)	69.8 ± 11.3 (*n* = 4)	27.4 ± 7.1 (*n* = 8)	NA	NA	NA	NA
Cavity volume (cm^3^)	360, 243 (*n* = 2)	Unconfirmed	354.1 ± 110.6 (*n* = 8)	NA	NA	NA	NA

Note

Unique *M*. *yanbarensis* roosts were pooled due to low sample size (*n* = 10). Only one record of the *M*. *yanbarensis* roost used as both a nonmaternity and maternity roost was included. Significance levels of Dunn's tests with Bonferroni correction are indicated with asterisks (*p* < .05*, <.01**, <.001***). DBH, cavity entrance area, and cavity volume were not statistically compared due to small sample sizes.

Abbreviations: M, maternity *M*. *ryukyuana* roosts; Myo, Myotis *yanbarensis* roosts; NM, nonmaternity *Murina ryukyuana*roosts.

### 3.1 Roost characteristics

**FIGURE 2:**
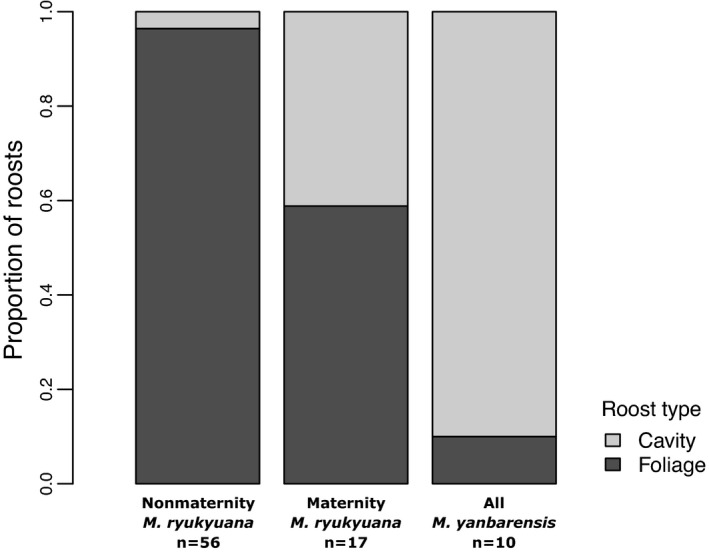
Proportion of roost types among unique roosts of *Murina ryukyuana* and *Myotis yanbarensis*. *Murina ryukyuana* roosts were divided into nonmaternity or maternity roosts

Solitary *M. ryukyuana* of all age classes and reproductive status utilized a variety of roost types and plant species as nonmaternity roosts (*n* = 56; Figure 2, Appendices S2 and S3). Nonmaternity roosts were most often in the dead or living foliage of understory plants, with some roosts in the fallen foliage of canopy species (Appendix S3). The foliage of many common trees was notably not used (e.g., *Castanopsis sieboldii* and *Pinus ryukyuana*), while the foliage of two common understory species, *Blechnum orientale* and *Alpinia intermedia* (Appendix S3A, B), represented 55% of all unique nonmaternity roosts (*n* = 10, 21, respectively; Appendix S2). Two small tree cavities were used as nonmaternity roosts.

*Murina ryukyuana* maternity roosts were found in a similar variety of plant species (*n* = 17; Appendices S2 and S4) but more often in cavities compared with nonmaternity roosts (Figure 2). All foliage maternity roosts were in stands ≥57 years old. Cavity maternity roosts were in stands ≥61 years old, and all but one were located in *Castanopsis sieboldii* (Appendix S2). Mean DBH of cavity maternity roosts was 22.8 ± 4.8 cm (*n* = 13). The mean minimum number of individuals per maternity roost ranged from 2 to 15 (mean 6 ± 1). Maternity roost entrances were larger (mean 70 cm^2^; *n* = 4) than for nonmaternity roosts (25 and 35 cm^2^), but we were unable to measure internal dimensions.

All ten unique *M. yanbarensis* roosts were in gulch bottoms, usually directly next to flowing water, and almost entirely in small tree cavities (Figure 2, Appendix S5). One nonmaternity roost included two adults, while all others were used by solitary individuals. Two nonmaternity roosts were in snags, one in a liana cavity, one in an overhanging root cavity, and one in a tree fern frond tube. A pregnant female roosted with 2–3 other individuals in an old Okinawa woodpecker (*Sapheopipo noguchi*) roost in an unidentifiable snag (Appendix S5B), and a lactating female roosted once with a fledged juvenile in a live *Toxicodendron succedaneum* cavity (Appendix S5C). DBH for all *M. yanbarensis* roosts was 18.4 ± 3.7 cm. Cavities were generally small; entrance area was 27.4 ± 7.1 cm^2^, and cavity volume was 354.1 ± 110.6 cm^3^. The first maternity roost was 844 cm^3^, but the second was too high to measure. *Myotis yanbarensis* roost height was 2.7 ± 0.5 m overall, and the maternity roosts were 2.0 and 4.5 m high, respectively.

The proportion of cavity roosts differed significantly (*p* < .001) between *M. ryukyuana* nonmaternity (4%), maternity roosts (41%), and *M. yanbarensis* roosts (90%; Figure 2). *Murina ryukyuana* maternity roosts were higher (4.3 ± 0.6 m) than *M. ryukyuana* nonmaternity roosts (2.3 ± 0.5 m), and on less southwesterly slopes (Table 2; *p* < .001 and .05, respectively). Stand ages for *M. ryukyuana* and *M*. *yanbarensis* maternity roosts were 20–35 years older than for *M*. *ryukyuana* nonmaternity roosts (51.9 ± 1.8 years old; *p* < .001). *Murina ryukyuana* maternity roosts were in forests with higher canopies than *M. ryukyuana* nonmaternity roosts (*p* < .01). Understory cover and stream distance were significantly lower at *M. yanbarensis* roost sites (51.5 ± 8.5% and 7.2 ± 3.9 m, respectively) than at *M. ryukyuana* roost sites (*p* values <.05 but vary). *Myotis yanbarensis* roost site understory cover ranged from 20% to 90%, with values <50% resulting from an absence of vegetation in the streambed. No roosts were found outside of forests, and canopy cover was uniformly high (mean >98% for all roost groups). *Murina ryukyuana* spent 1.6 ± 0.3 days per roost before switching. Maximum roost‐switch distance among *M. ryukyuana* (*n* = 17) was 178 ± 32 m (range 8–480 m). Roost fidelity was higher for *M. yanbarensis* (2.7 ± 0.5 days per roost) than *M. ryukyuana*, but only four *M. yanbarensis* were found in more than one roost. Maximum roost‐switch distance among *M. yanbarensis* (*n* = 4) was 701 ± 580 m (range 35–2,436 m).

### 3.2 Murina ryukyuana roost site selection

The only model supported by the data included canopy height, SWness, and stand age (ΔAIC_C_ ≤ 4; Table 1). Although we did not consider the global model ecologically relevant, the fact that it received 59% of the AICc weight suggests that our a priori hypotheses were not strongly supported by the data. The quadratic stand age term in the warm‐tall‐intermediate model was considered uninformative as it did not improve model fit (Arnold, 2010). According to the remaining warm‐tall‐old model, the likelihood of a site being used for roosting by *M. ryukyuana* increases 85% with each unit increment in SWness (1 unit = 90°), increases 235% with each meter increase in canopy height, and, contrary to our prediction, *decreases* 60% with each year increase in stand age (Table 3). Odds ratios were similar according to the discarded warm‐tall‐intermediate model, except that the likelihood of a site being used by *M. ryukyuana increased* 152% with each year increase in stand age. On average, SWness was 0.4 units (roughly 45˚) greater, canopy height 3 m taller, and stand age 10 years younger at *M. ryukyuana* roost sites than at random sites.

**TABLE 3:** Parameter estimates, st. errors (*SE*), odds ratios (OR), and 85% confidence intervals (CI) for the warm‐tall‐old model of roost selection by *Murina ryukyuana*

Parameter	Estimate	*SE*	Odds ratio	Upper 85%, OR CI	Lower 85%, OR CI
Intercept	−0.08	0.20			
SWness	0.62	0.21	1.85	1.38	2.54
Canopy height	1.21	0.27	3.35	2.33	5.06
Stand age	−0.91	0.23	0.40	0.29	0.56

### 3.3 Phenology

**FIGURE 3:**
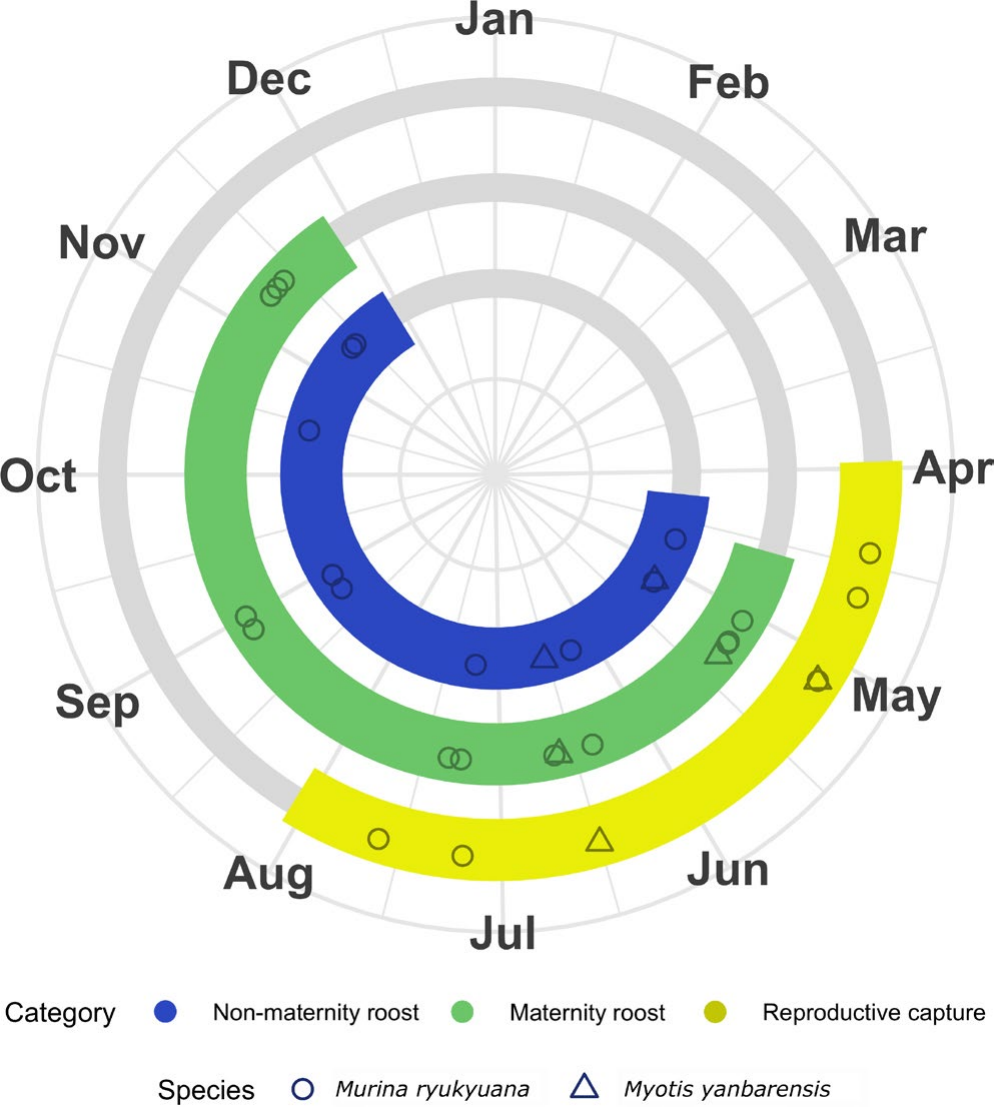
Circular plot showing phenological periods and records for adult female *Murina ryukyuana* (circles) and *Myotis yanbarensis* (triangles). Records for radio‐tracked bats represent the median date during each tracking period. Reproductive period (yellow) includes all capture records of pregnant or lactating females, and maternity roosts (green) include communal roosts of juveniles, reproductive females, or postlactating females. Postlactating bats are not included if roosts were not found. Periods include 14‐day buffers at the beginning and end. Includes data from this study (*n* = 17), Maeda et al., 2001 (*n* = 2), Funakoshi et al., 2019 (*n* = 12), and V. Dinets, unpublished (*n* = 1)

Capture and radio‐tracking records for adult female and juvenile *M. ryukyuana* from this and prior studies demonstrated a minimum reproductive period (pregnant or lactating) between 14 April and 20 July (Figure 3). *Murina ryukyuana* maternity roosts have been found from 2 May to 13 November. In this study, three adult female and three juvenile *M. ryukyuana* used maternity roosts (and sometimes solitary nonmaternity roosts) between 2 May and 12 July, while three postlactating females roosted only solitarily between 23 April and 22 October. The three reproductive females from this study were captured between 21 April and 7 July. All *M. yanbarensis* records were from this study. A pregnant *M. yanbarensis* was captured on 5 May and tracked to the same maternity roost on 11 and 12 May, while another maternity roost was used on 19 June by a lactating *M. yanbarensis* caught on 16 June.

## 4 DISCUSSION

Our radio‐tracking results represent the first descriptions of natural *M*. *ryukyuana* and *M*. *yanbarensis* maternity day roosts, as well as the first documented breeding on Okinawa. Our results support two of our primary hypotheses; both species roosted only in forests, and *M*. *ryukyuana* roosted in both foliage and cavities. *M*. *yanbarensis* roosted only in old‐growth forests, primarily in woody cavities but also in one tree fern frond tube. The degree to which *M*. *ryukyuana* maternity roosts are reliant on cavities and stands >50 years is unclear. *Myotis yanbarensis* showed a strong preference for roosting along gulch bottoms and streams, and both species roosted remarkably low off the ground throughout the year. Our analysis of roost site selection by *M*. *ryukyuana* was inconclusive, particularly regarding stand age, but suggests that the importance of southwesterly aspects and taller canopies should be investigated further. The poor fit of our models may be due to small sample sizes or biases in the data structure (e.g., pooling of both nonmaternity and maternity roost sites). We also may have missed an important variable; we suspect that understory cover may better explain *M*. *ryukyuana* roost site selection and recommend further investigation.

*Murina ryukyuana* in particular may be negatively impacted by understory foliage removal. Most roosts were only 1–2 m high, including three foliage maternity roosts, similar to examples from Tokunoshima and other *Murina* species (Fukui et al., 2012; Funakoshi et al., 2013, 2016). Though its diet is unknown, the low wing aspect ratio and faint frequency‐modulated echolocation of *M*. *ryukyuana* (Funakoshi et al., 2019; Norber & Rayner, 1987; Schnitzler et al., 2003), as well as the diet of congenerics (Ma et al., 2008; Schulz & Hannah, 1998), suggest that this species likely forages in clutter. Therefore, understory removal probably temporarily destroys both roosting and foraging habitats.

*Myotis yanbarensis* roosted primarily in woody cavities along gulch bottoms and streams through forests >70 years old. This roost specialization may be a limiting factor for *M*. *yanbarensis* populations that also raises the risk of extinction (Sagot & Chaverri, 2015). A handful of *M. yanbarensis* have been observed in tunnels (Asari et al., 2021) and rock crevices along streams (H. Tamura, personal communication), and these potentially important roosts warrant investigation. Many tree‐roosting bats prefer to roost near water, presumably to reduce commuting time to foraging grounds (Campbell, 2009; Kalcounis‐Rüppell et al., 2005). The higher wing aspect ratio of *M*. *yanbarensis* relative to *M*. *ryukyuana* and the relatively low understory cover around *M*. *yanbarensis* roosts suggest that *M*. *yanbarensis* prefers relatively uncluttered corridors within forests (Norber & Rayner, 1987). Dietary information would help clarify the foraging habitat of this species. On Amami Ōshima, individuals have been captured over roads through old‐growth forest (Asari & Kimoto, 2016; Funakoshi et al., 2019), and streambeds may simply be the only flyways through old‐growth forest on Okinawa.

Old‐growth forests appear to be important breeding habitats for both *M*. *ryukyuana* and *M*. *yanbarensis*. Only old‐growth forests were used by maternity colonies of both species even when tracked bats traveled through younger stands very close by. This preference for mature stands has been reported for other cavity‐roosting bats (e.g., Burgar et al., 2015; Perry et al., 2007) and may be related to higher cavity densities in older stands (Matsumoto et al., 2015). Also like other cavity‐roosting bats (e.g., Dietz et al., 2018), both *M*. *ryukyuana* and *M*. *yanbarensis* utilized old woodpecker cavities, and the recovery of woodpeckers in the central Ryukyu Archipelago bodes well for these bats (Kotowska et al., 2020). Our results are inconclusive as to the importance of roost tree diameter or cavity volume, but *M*. *ryukyuana* may utilize more cavity roosts during pup‐rearing or cold winter months for their insulating benefits (Klug et al., 2012).

Existing records suggest that pregnancy and lactation in *M*. *ryukyuana* and *M*. *yanbarensis* occur from April to at least mid‐July. Two reproductive female captures, ten maternity roosts, and three nonmaternity female roosts for *M*. *ryukyuana* were found in the literature. These included maternity roosts found in artificially placed leaves in August–November on Tokunoshima (Funakoshi et al., 2019), lactating females caught on Tokunoshima in late July (Maeda et al., 2001), and a night roost of mothers and infants seen 8 May on Amami Ōshima (V. Dinets, unpublished). The latter is the only record of prevolant *M*. *ryukyuana* and suggests that bats are heavily pregnant in April. Insect parts observed in the feces of juveniles indicated successful foraging by early June. However, continuing lactation suggests that offspring are reliant on mothers to some degree until at least mid‐July (Maeda et al., 2001). Juveniles and postlactating females have been observed together on Tokunoshima between August–November (Funakoshi et al., 2013, 2019), indicating that communal roosting continues beyond the lactation period. It is uncertain whether networks of related individuals are maintained throughout the breeding season, but fluctuating colony sizes and frequent roost‐switching in *M*. *ryukyuana* suggest a fission–fusion social system common among plant‐roosting bats (Patriquin & Ratcliffe, 2016). Records are far fewer for *M*. *yanbarensis*, but reproductive timing appears similar. Although roosts were not located, a postlactating female was captured in October (Preble et al., in press), and postlactating females were caught on Amami Ōshima in mid‐July (Asari & Kimoto, 2016).

We found too few roosts to explain the apparent rarity of *M*. *yanbarensis*. Based on our limited results, we surmise that *M*. *yanbarensis* occurs in lower numbers in a relatively restricted range due to a combination of greater roost specialization (mentioned previously) and a lower reproductive rate compared with *M*. *ryukyuana*. When perfect counting was possible (9 of 23 records), *M*. *ryukyuana* maternity colonies tended toward a ratio of one adult to two juveniles (e.g., Appendix S4C), suggesting that *M*. *ryukyuana* may birth 1–3 pups per year similar to other *Murina* species (Kuramoto & Uchida, 1981, Garbino et al., 2021). Conversely, our only record of an *M*. *yanbarensis* mother and pup (Appendix S5D) suggests that this species is monotocous like other *Myotis* species and most other Chiroptera (Racey & Entwistle, 2000). Differences in the current rarity of *M*. *ryukyuana* and *M*. *yanbarensis* may thus partially reflect differences in population growth following severe declines experienced during periods of large‐scale clear‐cutting such as the 1950s post‐WWII reconstruction period. The higher capture rate of *M*. *yanbarensis* on Amami Ōshima (Funakoshi et al., 2019; Preble et al., in press) may reflect the longer period since large‐scale clear‐cutting compared with Okinawa and the resulting larger areas of suitable old‐growth breeding habitat. Clarifying the extent of range restriction in *M*. *yanbarensis* and the ecological causes (e.g., habitat requirements and life history) should be a high priority for future research to avoid inadvertently destroying critical habitat.

### 4.1 Management implications

Given their roosting habits and the lack of evidence of other threats, forest degradation is likely the greatest threat to *M*. *ryukyuana* and *M*. *yanbarensis* (Fukui & Sano, 2019a, 2019b). Local forestry guidelines already attempt to mitigate impacts to other threatened taxa and could be updated to also reduce negative impacts to bats. Based on our results, we make the following suggestions:

1. Retain understory vegetation to preserve *M*. *ryukyuana* habitat and to avoid disturbing the low roosts of both bat species. Understory removal in Yambaru is already of conservation concern due to adverse impacts on native biodiversity (Azuma et al., 1997; Ito et al., 2000).
2. Riparian trees, including mature trees and snags, should not be removed, particularly in old‐growth forests, given the rarity of *M*. *yanbarensis* and its apparent reliance on these habitats. Local forestry guidelines already recommend retaining riparian corridors for wildlife, but currently only advise against removing undergrowth and small diameter trees (Okinawa Prefecture Department of Agriculture, Forestry, & Fisheries, 2013). Riparian exclusion zones are a common forestry practice for maintaining biodiversity, including bats (Lloyd et al., 2006, Law et al., 2016), and the natural succession of riparian trees likely produces more snag cavities than thinning (Pollock & Beechie, 2014).
3. Old‐growth forest (especially >70 years old) should be preserved to provide ample cavity roosts, and some mature trees and snags should be retained in harvested or thinned forests. Although the exact cavity densities required by each species are unknown, both bat species formed small maternity colonies and switched roosts regularly, suggesting that they require high roost availability (Russo et al., 2005).
4. To avoid disturbing maternity roosts, trees and understory foliage should not be removed between April and mid‐July, when bats are pregnant or lactating. Current forestry guidelines advise against harvesting during the Okinawa woodpecker breeding season between March and June (Okinawa Prefecture Department of Agriculture, Forestry, & Fisheries, 2013). Extending this period through July would be a relatively simple way to accommodate the area's endangered plant‐roosting bats.

We are cautiously optimistic about the outlook of *M*. *ryukyuana* and *M*. *yanbarensis* given current low levels of logging and increasing interest in sustainable management across their range. Still, further research is needed, particularly concerning *M*. *yanbarensis*, and these species should be incorporated into forest management plans rather than just listed as threatened. We hope that this study serves as a reference for both local conservation planning and further roosting ecology research in Asia.

## CONFLICT OF INTEREST

We have no competing interests to declare.

## AUTHOR CONTRIBUTION

**Jason H. Preble:** Conceptualization (equal); Data curation (lead); Formal analysis (lead); Funding acquisition (equal); Investigation (lead); Methodology (equal); Visualization (lead); Writing‐original draft (lead); Writing‐review & editing (lead). **Christian E. Vincenot:** Conceptualization (equal); Funding acquisition (equal); Methodology (equal); Resources (equal); Supervision (lead); Writing‐review & editing (supporting). **Kazuhiko Saito:** Data curation (equal); Formal analysis (supporting); Investigation (supporting); Writing‐review & editing (supporting). **Nobuhito Ohte:** Funding acquisition (equal); Resources (equal); Supervision (supporting); Writing‐review & editing (supporting).

## ETHICAL APPROVAL

This research was carried out under permits from Aha Dam Office, Kunigami Village Office, University of the Ryukyus Experimental Forest Yona Field, Okinawa Regional Forest Office, Okinawa Prefectural Government Hokubu Agriculture, Forestry and Fisheries Promotion Center, and the Japanese Ministry of the Environment. Animal research was also permitted by Kyoto University (permits Inf‐K17008 and Inf‐K19007).

## Supporting information

Figure S1

Figure S2

Figure S3

Appendix S1‐S5

## Data Availability

Raw data and R scripts are available on Figshare (https://doi.org/10.6084/m9.figshare.16528209).
