# Supplementary figures and images for: Roosting ecology of endangered plant‐roosting bats on Okinawa Island: Implications for bat‐friendly forestry practices
Source: Ecol Evol. 2021 Sep 17;11(20):13961–71. doi: 10.1002/ece3.8101 (PMC8525085; doi:10.1002/ece3.8101)

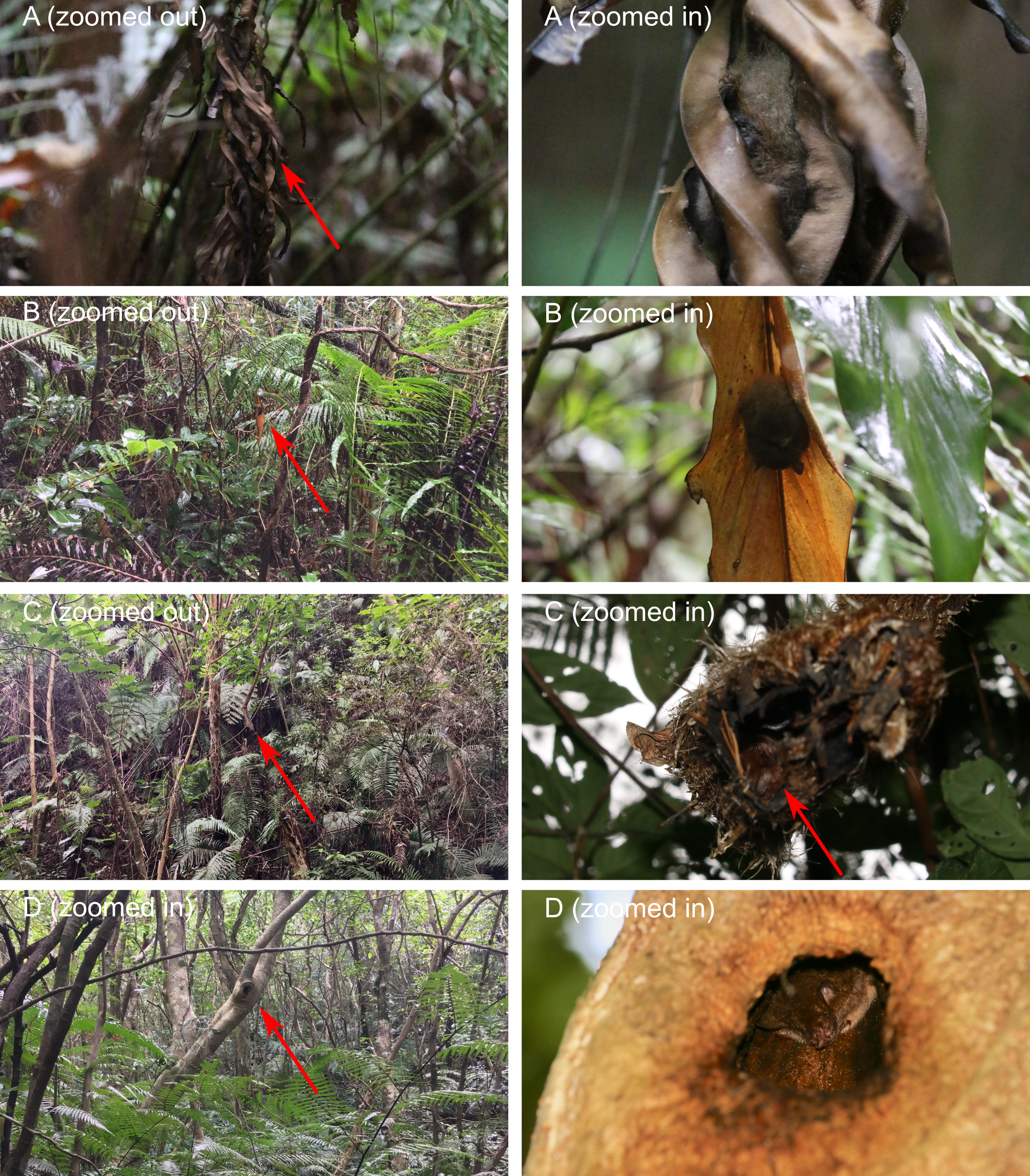

Supplement: Supplementary file 1 — Figure S1 [file ECE3-11-13961-s002.tif]

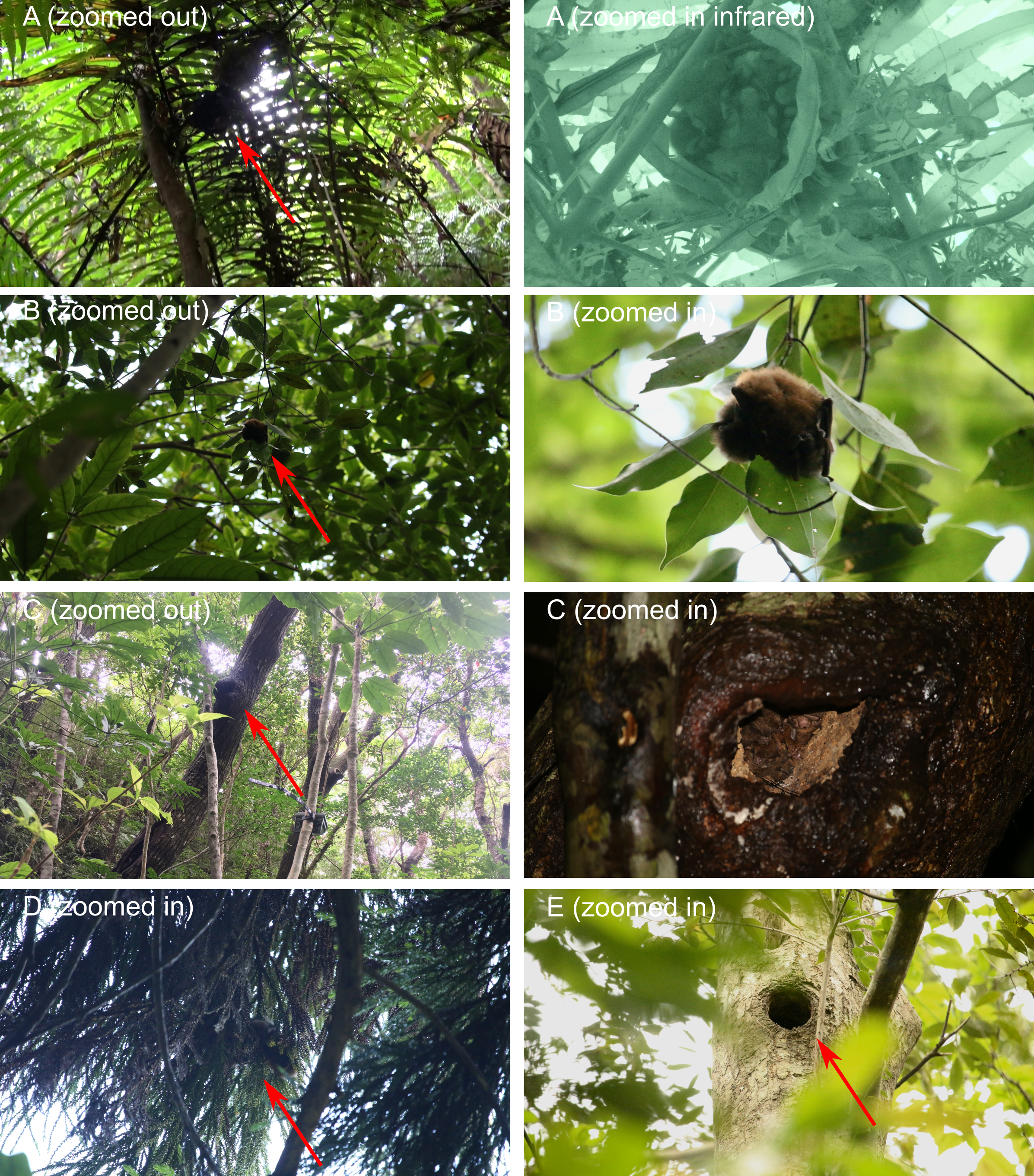

Supplement: Supplementary file 2 — Figure S2 [file ECE3-11-13961-s001.tif]

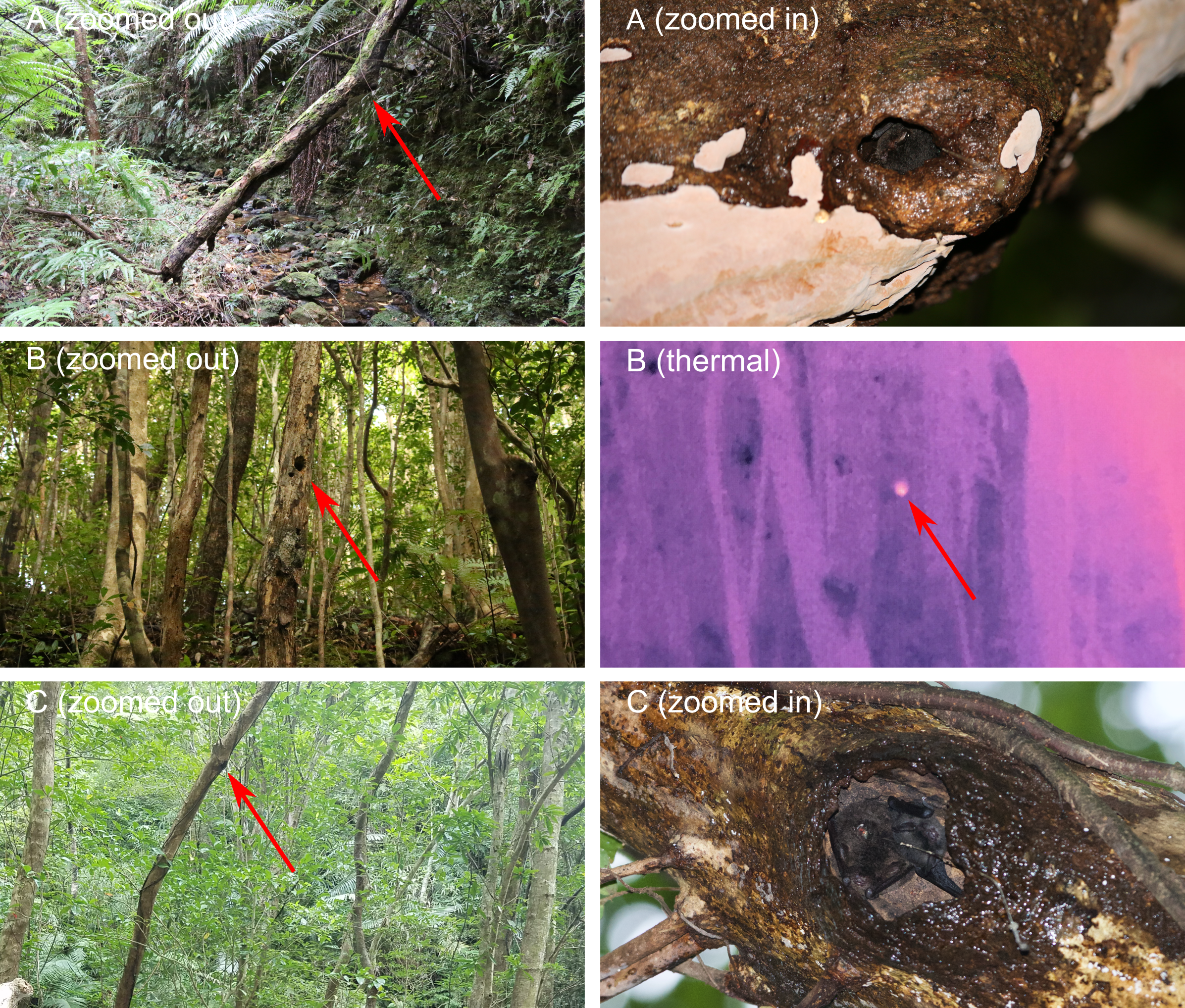

Supplement: Supplementary file 3 — Figure S3 [file ECE3-11-13961-s004.tif]
