# Supplementary material for: Roosting ecology of endangered plant‐roosting bats on Okinawa Island: Implications for bat‐friendly forestry practices
Source: Ecol Evol. 2021 Sep 17;11(20):13961–71. doi: 10.1002/ece3.8101 (PMC8525085; doi:10.1002/ece3.8101)
Supplement: Supplementary file 4 — Appendix S1‐S5 [file ECE3-11-13961-s003.docx]

**Appendix S1.** Radio-tracked *Murina ryukyuana* and *Myotis yanbarensis*. Total indicates the number of times bat’s roost location was confirmed (visually) or unconfirmed (location only triangulated). Roost fidelity calculated as mean number of days before switching. Totals listed for all fields except for roost fidelity and maximum distance between consecutive roosts (max switch dist), for which mean ± st. error are listed. M = male; F = female; A = adult male; J = juvenile; P = pregnant; L = lactating. Note that total number of unique roosts may be less than sum for each individual if multiple individuals used the same roost. Radio-transmitters were also attached to four other *M. yanbarensis* that were not relocated.

|  |  |  | |  | |  | |  | |  | **# Unique roosts** | |  |  |
| --- | --- | --- | --- | --- | --- | --- | --- | --- | --- | --- | --- | --- | --- | --- |
| **Species** | **Bat ID** | **Sex** | **Repro status** | | **Month** | | **Maternity** | | **Total records** | | **Conf** | **Unconf** | **Fidelity** | **Max switch dist (m)** |
| *M. ryukyuana* | MU01 | M | Adult | | Sep, Oct | | N | | 17 | | 5 | 12 | 1.1 | 290 |
|  | MU02 | M | Adult | | Oct | | N | | 17 | | 6 | 8 | 1.1 | 480 |
|  | MU03 | F | Post-lactating | | Oct | | N | | 17 | | 5 | 11 | 1.0 | 192 |
|  | MU24 | M | Adult | | Mar | | N | | 10 | | 2 | - | 5.0 | 140 |
|  | MU25 | M | Adult | | Mar | | N | | 6 | | 3 | - | 1.5 | 185 |
|  | MU26 | M | Adult | | May | | N | | 7 | | 2 | - | 3.5 | 12 |
|  | MU27 | M | Adult | | May | | N | | 9 | | 7 | 1 | 1.1 | 154 |
|  | MU28 | M | Adult | | May, Jun | | N | | 6 | | 2 | 1 | 3.0 | 75  9  40  124 |
|  | MU29 | F | Lactating | | Jul | | Y | | 2 | | 2 | - | 1.0 | 8 |
|  | MU30 | F | Juvenile | | Jul | | Y | | 2 | | 2 | - | 1.0 | 40 |
|  | MU31 | F | Post-lactating | | Aug | | N | | 4 | | 4 | - | 1.0 | 124 |
|  | MU32 | M | Adult | | Aug, Sep | | N | | 11 | | 8 | 2 | 1.1 | 157 |
|  | MU33 | F | Post-lactating | | Aug, Sep | | N | | 7 | | 6 | - | 1.2 | 106 |
|  | MU49 | F | Pregnant | | Apr, May | | Y | | 4 | | 2 | - | 2.0 | 387 |
|  | MU55 | F | Lactating | | May | | Y | | 4 | | 4 | - | 1.0 | 175 |
|  | MU60 | F | Juvenile | | Jun | | Y | | 11 | | 8 | 1 | 1.1 | 342 |
|  | MU66 | F | Juvenile | | Jun | | Y | | 7 | | 5 | - | 1.2 | 150 |
| Total/Average | 17 | 8M, 9F | 8A, 3J, 1P, 2L, 3PL | |  | | 6 Mat. | | 141 | | 73 | 36 | 1.6±0.3 | 178±32 |
| *M. yanbarensis* | MY01 | M | Adult | | Feb | | N | | 1 | | 1 | - | NA | NA |
|  | MY02 | M | Adult | | Feb, Mar | | N | | 7 | | 2 | - | 3.5 | 35 |
|  | MY07 | M | Adult | | Oct | | N | | 7 | | 2 | - | 3.5 | 101 |
|  | MY14 | F | Pregnant | | May | | Y | | 3 | | 2 | - | 1.5 | 2436 |
|  | MY16 | F | Lactating | | Jun | | Y | | 2 | | 1 | - | NA | NA |
|  | MY18 | M | Adult | | Jul | | N | | 7 | | 3 | - | 2.3 | 231 |
| Total/Average | 6 | 4M, 2F | 4A, 1P, 1L | |  | | 2 Mat. | | 27 | | 10 | 0 | 2.7±0.5 | 701±580 |

**Appendix S2.** Number of unique non-maternity (NM) and maternity (M) roosts found per plant species used by *Murina ryukyuana* and *Myotis yanbarensis*. Note total *M. yanbarensis* roosts exclude double-counting of one roost used as both NM and M.

|  | ***Murina ryukyuana*** | | | | ***Myotis yanbarensis*** | | | |
| --- | --- | --- | --- | --- | --- | --- | --- | --- |
| **Plant species** | **Total** | **NM** | **M** | **Roost description** | **Total** | **NM** | **M** | **Roost description** |
| *Actinidia rufa* | 0 | 0 | 0 | - | 1 | 1 | 0 | Cavity in liana |
| *Alpinia intermedia* | 10 | 10 | 0 | Dead foliage | 0 | 0 | 0 | - |
| *Blechnum orientale* | 24 | 21 | 3 | Dead foliage mostly, living foliage | 0 | 0 | 0 | - |
| *Castonopsis sieboldii* | 6 | 0 | 6 | Cavities, three woodpecker cavities | 1 | 1 | 0 | Cavity in overhanging exposed roots |
| *Cibotium barometz* | 4 | 4 | 0 | Living foliage | 0 | 0 | 0 | - |
| *Cinnamomum yabunikkei* | 0 | 0 | 0 | - | 1 | 1 | 0 | Tree cavity |
| *Cryptomeria japonica* | 4 | 2 | 2 | Foliage | 0 | 0 | 0 | - |
| *Cyathea lepifera* | 7 | 6 | 1 | Dead foliage, M in living foliage | 1 | 1 | 0 | Fern tube |
| *Dicranopteris linearis* | 1 | 0 | 1 | Dead foliage within thicket | 0 | 0 | 0 | - |
| *Elaeocarpus japonicus* | 2 | 0 | 2 | Living foliage and cavity | 0 | 0 | 0 | - |
| *Fatsia japonica var. liukiuensis* | 3 | 3 | 0 | Dead foliage | 0 | 0 | 0 | - |
| *Ficus benguetensis* | 1 | 1 | 0 | Dead foliage | 0 | 0 | 0 | - |
| *Lithocarpus edulis* | 1 | 1 | 0 | Dead foliage | 0 | 0 | 0 | - |
| *Machilus japonica* | 1 | 1 | 0 | Tree cavity | 0 | 0 | 0 | - |
| *Pleioblastus linearis* | 3 | 2 | 1 | Living foliage | 0 | 0 | 0 | - |
| *Schefflera heptaphylla* | 2 | 2 | 0 | Dead foliage, tree cavity | 0 | 0 | 0 | - |
| *Styrax japonica* | 0 | 0 | 0 | - | 1 | 1 | 0 | Tree cavity |
| *Tarenna gracilipes* | 1 | 0 | 1 | Living foliage | 0 | 0 | 0 | - |
| *Tetradium glabrifolium var. glaucum* | 0 | 0 | 0 | - | 1 | 1 | 0 | Tree cavity |
| *Toxicodendron succedaneum* | 0 | 0 | 0 | - | 1 | 1 | 1 | Cavity (same roost used as NM & M) |
| Unidentified | 3 | 3 | 0 | Dead foliage | 3 | 2 | 1 | Snag cavity, M in woodpecker cavity |
| **Total** | **73** | **56** | **17** |  | **10** | 8 | 2 |  |

**Appendix S3.** Examples of roosts used by solitary non-maternity *Murina ryukyuana*. (A) Dead frond of *Blechnum orientale*. (B) Dead leaf of *Alpinia intermedia* (C) Within tube of dead *Cyathea lepifera* frond. (D) Tree cavity in *Elaeocarpus japonicus*.

**Appendix S4.** Examples of maternity roosts used by *Murina ryukyuana*. (A) Living frond of *Blechnum orientale.* (B) Mother and two juveniles (note grey pelt) in living foliage of *Tarenna gracilipes*. (C) Tree cavity in *Castonopsis sieboldii*. (D) *Cryptomeria japonica* foliage. (E) Old *Sapheopipo noguchii* nest cavity in *Castonopsis sieboldii*.

**Appendix S5.** Examples of roosts used by *Myotis yanbarensis*. (A) Non-maternity roost in small cavity of tree fallen over stream. Tree too degraded to identify to species. (B) First reported roost of pregnant females. Roost is an old *Sapheopipo noguchii* nest cavity. Tree too degraded to identify to species. Unable to take photo within cavity, but thermal image on right shows body heat of bats escaping from roost entrance. (C) First reported roost of a mother (transmitter attached) with child (dark grey pelt). Roost is a cavity in *Toxicodendron succedaneum.*
